# Human Infection Caused by *Clostridium hathewayi*

**DOI:** 10.3201/eid1011.040006

**Published:** 2004-11

**Authors:** Sameer Elsayed, Kunyan Zhang

**Affiliations:** * University of Calgary, Calgary, Alberta, Canada;; † Calgary Laboratory Services, Calgary, Alberta, Canada

**Keywords:** Clostridium hathewayi, human infection, case report, hepatic abscess, bacteremia, dispatch

## Abstract

We describe a 27-year-old man with acute cholecystitis, hepatic abscess, and bacteremia caused by *Clostridium hathewayi*, a newly described gram-negative, endospore-forming, rod-shaped bacterium. This report is the first of human infection caused by this microorganism.

The genus *Clostridium* is a phylogenetically heterogeneous group of anaerobic, endospore-forming, rod-shaped bacteria; they are usually gram positive, but some species may stain gram variable or gram negative (*1**,**2*). *Clostridium* strains are widely distributed in the environment and form part of the normal colonic microflora of humans and many animals (*1**,**2*). More than 150 species have been described to date, but most are believed to be harmless saprophytes (*1*). Pathogenic *Clostridium* spp. may be involved in a wide variety of human infections or illnesses. Such conditions are usually endogenous (e.g., brain abscess, pneumonia, intrabdominal abscess, cholecystitis, bacteremia) and arise from the host's own microflora; other illnesses may be exogenous (e.g., food poisoning, pseudomembranous colitis, tetanus, botulism, myonecrosis) (*1*). The most commonly encountered, clinically important clostridia include *Clostridium perfringens*, *C. clostridioforme*, *C. ramosum*, *C. butyricum*, *C. innocuum*, *C. septicum*, *C. tertium*, and *C. difficile* (*1*). *C. hathewayi* is a newly described species that was first reported by Steer et al. (*3*). The species was named after Charles L. Hatheway in recognition of his contributions to the *C. botulinum* group of organisms (*3*).

## Case Report

Our patient was a 27-year-old, previously healthy Chinese man who immigrated to Canada 10 years previously. The patient sought treatment at the hospital for a 2-week history of intermittent fever and chills that were associated with sharp, nonradiating, right upper quadrant abdominal pain. He did not report nausea, vomiting, diarrhea, or jaundice. The patient did not use alcohol heavily, use intravenous drugs, or engage in high-risk sexual activity. The patient did not have a history of recent travel outside of Canada and was not taking any medications. His medical history and family history were unremarkable. Upon physical examination, the patient appeared mildly ill and had a temperature of 39.8°C, along with sinus tachycardia. Other findings were unremarkable except for mild right upper quadrant abdominal discomfort with deep palpation. Laboratory testing showed a peripheral leukocytosis with a left shift. Abdominal ultrasound and computed tomographic (CT) scans showed gallbladder wall thickening and inflammation, multiple gallstones, and a single large fluid collection in the right lobe of the liver, with multiple adjacent smaller satellite fluid collections. Acute cholecystitis and hepatic abscess were diagnosed. Two sets of BacT/Alert FAN (bioMerieux Inc., Durham, NC) aerobic and anaerobic blood cultures were drawn, after which the patient received empiric intravenous piperacillin-tazobactam therapy.

**Figure 1:**
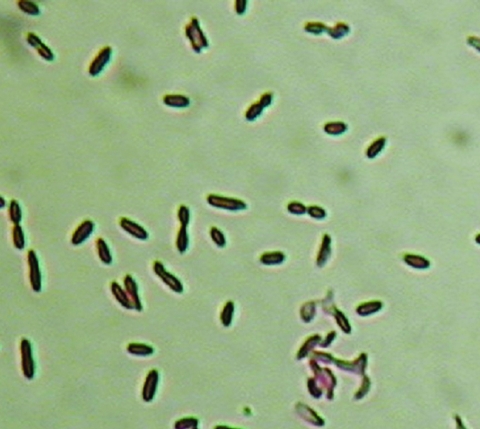
Gram strain of *Clostridium hathewayi* from growth on solid agar media. Note the gram-negative staining characteristics and presence of subterminal endospores. Magnification 1,000x.

A large liver abscess was drained by using CT scans as a guide; samples of the liver abscess were submitted for aerobic and anaerobic culture. Gram stain of abscess fluid showed heavy neutrophils and gram-negative rods. After 48 hours of incubation in the BacT/Alert 3D system (bioMerieux, Inc.), the anaerobic bottles from both sets of blood cultures were positive for similar, gram-negative, rod-shaped bacteria. The blood and abscess isolates did not grow aerobically, although anaerobic growth on brucella blood agar media (PML Microbiologicals, Wilsonville, OR) showed identical-looking, nonhemolytic, motile organisms, with colonial and microscopic morphologic features typical of *Clostridium* spp. However, endospores were not visualized initially, and the organisms persistently stained gram negative. Growth was observed on anaerobic phenylethyl alcohol agar; attempts to grow the organisms on kanamycin-vancomycin laked blood and bacteroides bile esculin agars (PML Microbiologicals) failed. Both isolates demonstrated sensitivity to vancomycin (5 µg) and kanamycin (1,000 µg) and resistance to colistin (10 µg) special-potency identification discs. Tests for catalase, indole, lecithinase, lipase, and reverse Christie-Atkins-Munch-Peterson were negative. Since routine conventional phenotypic identification algorithms were not successful in identifying organisms, the isolates initially underwent partial 16S ribosomal RNA (rRNA) gene sequencing using MicroSeq 500 kits and an ABI Prism 3100 Genetic Analyzer (Applied Biosystems, Foster City, CA). Full-length sequencing of the 16S rRNA gene was subsequently performed for more definitive identification. The blood and abscess isolates had identical sequences; a GenBank BLAST (http://www.ncbi.nlm.nih.gov/BLAST/) search showed a 99% match of their full-length 16S rRNA gene profiles with those of a previously characterized strain of *C. hathewayi* (GenBank accession no. AJ311620). Repeat subculture and Gram stain of the organism showed the presence of gram-negative rods with subterminal endospores (Figure 1).

The isolates were susceptible to penicillin G (MIC = 0.064 µg/mL), clindamycin (MIC = 0.064 µg/mL), and metronidazole (MIC = 0.032 µg/mL) by Etest. Despite intense efforts, no other microorganisms were isolated from the blood or liver abscess specimens. Antimicrobial therapy was subsequently switched to intravenous ceftriaxone and metronidazole. After marked clinical improvement, the patient was discharged home on oral therapy.

## Conclusions

**Figure 2:**
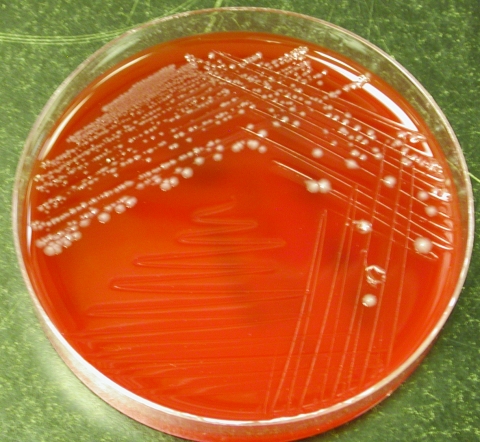
Colonial morphology of *Clostridium hathewayi* on brucella blood agar media after 48 hours of anaerobic incubation.

Similar to certain members of the genus *Clostridium* (e.g., *C. clostridioforme* and *C. ramosum*), the organism has a propensity to stain gram negative (*3*). Subterminal, oval-to-round endospores may be visible. Strains may or may not be motile. Colonies are usually 3 mm in diameter after 72 hours of incubation and are nonhemolytic, grayish-white, opaque, convex, round, shiny, and have a slightly irregular margin (*3*), similar to our observations (Figure 2). Isolates are typically saccharolytic and ferment a variety of carbohydrate compounds but fail to hydrolyze gelatin or urea, reduce nitrate, or produce indole, lecithinase, or lipase (*3*). Although these phenotypic tests can possibly distinguish this organism from closely related species, these methods are too cumbersome and time-consuming for routine use in the clinical setting. However, the increasing ease, availability, and affordability of identification methods that use DNA sequencing have identified uncommon microbial pathogens that are difficult to identify and often encountered in the clinical microbiology laboratory. The 16S rRNA gene (≈1,500 bp in size) is ubiquitous in all eubacteria and has served as the principal target of bacterial identification protocols that are sequence-based. Each unique bacterial species has a distinctive 16S rRNA gene sequence profile (signature); hence, the signatures of unknown bacteria can then be compared to publicly available or commercial sequence databases to determine if the organism belongs to a particular known species.

**Figure 3:**
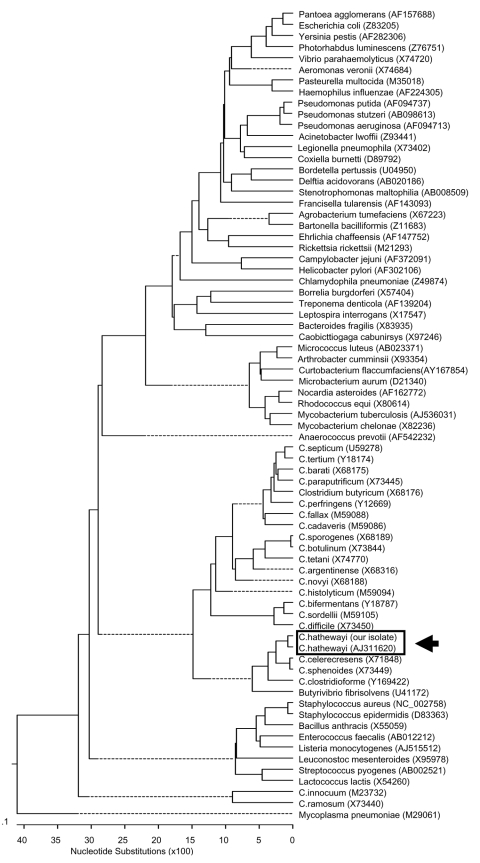
Phylogenetic tree showing the 16S rRNA relationships of our *Clostridium hathewayi* isolate (GenBank accession no. AY552788) wth various *Clostridium* species and other medically important bacteria. The tree was constructed by Clustal W analysis (DNASTAR Inc., Madison, WI), based on the entire 16S rRNA gene. The sequences were obtained from the GenBank database with their nucleotide sequence accession numbers in brackets. *Mycoplasma pneumoniae* was used as the outgroup to root the tree. Our *C. hathewayi* isolate and the published *C. hathewayi* strain (GenBank accession no. AJ311620) are boxed and delineated with an arrow.

Although the sequence profile of our isolate (GenBank accession no. AY552788) was 99% identical to the type strain of *C. hathewayi* reported by Steer et al. (*3*) (GenBank accession no. AJ311620), the two sequences are believed to be completely identical since the 1% difference in the sequence profiles was attributed to uncharacterized bases in the sequence reported by Steer. *C. hathewayi* displays the closest phylogenetic relationships with *C. celerecresens* and *C. sphenoides* (Figure 3), which may be found as part of the normal human colonic microflora (*3**,**4*). However, the natural habitat of *C. hathewayi* is not known. To the best of our knowledge, human infection caused by this bacterium has not been previously reported. The two isolates described by Steer et al. (*3*) were recovered from a phytic acid-degrading, organism enriching chemostat that had been injected with human feces from healthy donors. In contrast, the isolation of *C. hathewayi* from our patient's blood and hepatic abscess fluid specimens is convincing evidence of its clinical importance, although this finding needs to be corroborated by animal studies. The finding is not surprising, given that *Clostridium* spp. are not uncommonly implicated in cases of acute cholecystitis and associated bacteremia (*5*). Our report highlights the importance of *C. hathewayi* as a potential human pathogen.
